# Coenzyme Q10 and Melatonin for the Treatment of Male Infertility: A Narrative Review

**DOI:** 10.3390/nu14214585

**Published:** 2022-11-01

**Authors:** Gianpaolo Lucignani, Letizia Maria Ippolita Jannello, Irene Fulgheri, Carlo Silvani, Matteo Turetti, Franco Gadda, Paola Viganò, Edgardo Somigliana, Emanuele Montanari, Luca Boeri

**Affiliations:** 1 Department of Urology, Foundation IRCCS Ca’ Granda, Ospedale Maggiore Policlinico, 20122 Milano, Italy; 2 Department of Vascular Surgery, Foundation IRCCS Ca’ Granda, Ospedale Maggiore Policlinico, 20122 Milano, Italy; 3 Department of Gynecology and Obstetrics, Foundation IRCCS Ca’ Granda, Ospedale Maggiore Policlinico, 20122 Milan, Italy; 4 Department of Clinical Sciences and Community Health, University of Milan, 20122 Milan, Italy

**Keywords:** coenzyme Q10, melatonin, male reproduction, oxidative stress, supplementation

## Abstract

Background: Lifestyle and environmental factors can negatively impact fertility by means of oxidative stress. In this context, antioxidant supplementation therapy has gained much interest in recent years, and different molecules, alone or in combination, have been studied. Objective: The purpose of the present review is to investigate the evidence regarding the efficacy of coenzyme Q10 (CoQ10) and melatonin on male infertility. Methods: A literature search using PUBMED database from 2000 to October 2022 was performed to explore the role of CoQ10 and melatonin on male reproductive function. Conclusions: The analysis involved a narrative synthesis. CoQ10, alone or in combination, appears to reduce testicular oxidative stress and sperm DNA fragmentation and to improve sperm parameters; particularly sperm motility. Moreover, CoQ10 treatment is associated with higher pregnancy rates, both naturally and through assisted reproductive technology (ART). Larger studies are needed to precisely determine its clinical efficacy. Melatonin is a known antioxidant and preclinical studies have shown its ability to modulate reproductive function through hormonal and immune system regulation and sperm cell proliferation. Regardless, clinical studies are necessary to assess its potential in male infertility.

## 1. Introduction

Infertility, which is defined as the inability to conceive after at least 12 months of regular, unprotected sexual activity, is estimated to affect between 8 and 12 percent of reproductive-aged couples [1,2]. Male factor infertility (MFI) can be identified in around 50% of cases in this scenario [1,3]. Couple’s infertility is a frequent ailment, and the Global Burden of Disease survey found that, over the past three decades (1990–2017), the age-standardized prevalence of infertility has increased annually by 0.29% in males and 0.35% in women [4].

In the context of male infertility, causes can be classified as (I) congenital (II) acquired and (III) idiopathic, which comprises nearly 30% of infertile couples [5]. Idiopathic infertility is assumed to be associated with risk factors that have a detrimental impact on the reproductive capacity of the male population [6,7,8,9]. Oxidative stress, which is known to play a pathogenic role in a variety of diseases [10,11], may also have a relevant impact on spermatozoa’s activity. Hence, lifestyle factors such as smoking, alcohol use, obesity, varicocele, infections and psychological stress, which have been associated with infertility and poor sperm quality [12,13,14] may indeed exploit their effect through oxidative stress [15,16,17]. The process is thought to influence between 30 and 80% of subfertility cases, and, for this reason, the category of MOSI (male oxidative stress infertility) was created [18]. In this regard, it was suggested that increased oxidative activity may negatively impact the reproductive function by reducing sperm concentration and motility [19]. Accordingly, infertile men display imbalance of the blood oxidation status and the level of reactive oxygen species (ROS) in seminal fluid has been correlated with sperm motility, morphology, and count in astheno- and oligoastheno-teratospermic males [20]. Moreover, reports have linked these factors to sperm DNA fragmentation (SDF), a recurrent disruption observed in idiopathic male infertility [21,22]. As a proof of concept, several authors have investigated the efficacy of antioxidant supplementation (such as L-carnitine, selenium, Coenzyme Q10, ubiquinol, and vitamins C and E) in infertile men, with positive results on sperm quality [23]. However, the limited understanding of MOSI’s etiology has prevented the development of established guidelines regarding its treatment [24].

Coenzyme Q10 (CoQ10) is a fat-soluble ubiquinone with intracellular antioxidant activity, the lack of which has been observed in various sub sub-fertile conditions, (e.g., varicocele, oligozoospermia) [25]. Besides oxidative status balance, the substance is primarily involved in mitochondrial activity and energy-dependent processes, such as sperm motility.

Melatonin is a hormone secreted by the pineal gland of the brain [26], whose production is enhanced by darkness, and it is the primary regulator of circadian rhythms. In addition, melatonin and its metabolites act as free radical scavengers, hence protecting cells from oxidative stress, which could have a significant impact on infertility [27].

The purpose of this narrative review is to summarize and analyze the role of CoQ10 and melatonin in the treatment of male infertility.

## 2. Materials and Methods

The PubMed database was employed for the research. Studies were identified using combinations of the search terms “male reproduction”, “sperm”, “testis”, “gonad”, “fertility”, “semen quality” and “sex hormones”, in combination with “coenzyme Q10”, “melatonin” and “coenzyme”. We decided to limit the search to articles published from January 2000 to October 2022, in order to guarantee the relevance and currency of the paper. Only publications in English were included. Selection criteria included all published randomized controlled trials (RCTs) and non-randomized studies (NRSs) (e.g., observational, prospective, retrospective cohort studies, case-control studies), focusing on CoQ10 and melatonin and male reproductive function. Only clinical studies were considered. Articles identified through references were also included and analyzed. Original and review articles were included. After removing duplicates and papers not relevant to the topic of this article, we identified 61 papers to be included in this narrative review.

The narrative review checklist is reported in the Supplementary Table S1.

## 3. Results

Results from the retrieved articles are presented in subsections based on the type of treatment.

### 3.1. Coenzyme Q10

#### 3.1.1. CoQ10 Monotherapy

Based on previous pilot studies, Balercia et. al. [28] investigated the effect of daily 200 mg CoQ10 administration in a randomized, double-blind, placebo-controlled trial conducted on 28 infertile men and 27 control patients. After 6 months, seminal fluid analysis showed improvements in total and forward motility (33.14 ± 7.12% to 39.41 ± 6.80%, *p* < 0.0001, and 10.43 ± 3.52% to 15.11 ± 7.34%, *p* < 0.0003, respectively). Moreover, a cause–effect relationship was suggested by the finding of a significant increase in CoQ10 levels both in seminal fluid plasma and sperm cells [28].

In 2009, a larger RCT (106 subjects vs. 106 controls) investigated the efficacy of a daily 300 mg regimen of CoQ10 in infertile men with idiopathic oligoasthenoteratospermia (iOAT) [29]. After 26 weeks, higher total sperm counts (47.8 ± 11.2 × 10^6^ vs. 57.6 ± 14.4 × 10^6^, *p* = 0.01) and motility (23.1 ± 2.1% vs. 27.6 ± 2.2%, *p* = 0.01) were found in the CoQ10 group. Notably, the CoQ10 group showed a significant increase in inhibin B levels and a significant decrease in follicle stimulating hormone (FSH) values. Blood and seminal plasma CoQ10 were raised after treatment, and the latter strongly correlated with sperm count (r = 0.77, *p* = 0.01), sperm motility (r = 0.76, *p* = 0.01) and sperm morphology (r = 0.54, *p* = 0.02).

The same group subsequently studied the effect of a 26-week course of 200 mg daily CoQ10 vs. placebo. Compared to placebo, sperm density (16.8 ± 4.4 × 10^6^/mL vs. 28.7 ± 4.6 × 10^6^/mL, *p* = 0.005), motility (25.4 ± 2.1% vs. 35.8 ± 2.7%, *p* = 0.008) and morphology (14.8 ± 4.1% vs. 17.6 ± 4.4%, *p* = 0.01) were significantly higher in the treatment group, showing a positive correlation with treatment duration. Moreover, the increase in sperm density and motility remained evident even 3 months after treatment, though less markedly [30].

Another group later examined CoQ10′s potential in varicocele-related male infertility. After 3 months of 100 mg CoQ10 daily supplementation, Festa et. al. reported a significant improvement in sperm density (35.5 ± 3.4 × 10^6^/mL vs. 42.6 ± 4.5 × 10^6^/mL, *p* = 0.03), forward motility (20.1 ± 4.5% vs. 28.4 ± 4.9%, *p* = 0.03) and seminal plasma total antioxidant capacity (TAC) (106.6 ± 8.7 s vs. 148.4 ± 12.6 s, *p* < 0.01) [31].

On the other hand, an RCT by Nadjarzadeh did not observe a significant impact of a 12-week course of daily 200 mg CoQ10 on sperm parameters, although CoQ10 significantly increased seminal plasma’s TAC [32].

Investigating a dose response effect of CoQ10 integration on semen parameters, an RCT from 2019 by Alahmar et al. observed a stronger effect of a 3-month 400 mg regimen, compared to the standard 200 mg, on progressive and total motility and TAC [33].

Two years later, the same group compared the efficacy of CoQ10 and selenium on men affected by iOAT. Coenzyme Q10 again proved to positively affect sperm concentration (8.22 ± 6.88 × 10^6^/mL vs. 12.53 ± 8.11 × 10^6^/mL, *p* < 0.01) and motility (progressive motility 16.54 ± 9.26% vs. 22.58 ± 10.15%, *p* < 0.01; total motility 25.68 ± 6.41% vs. 29.96 ± 8.09%, *p* < 0.01) as well as TAC (1.1 ± 0.30 mmol/L vs. 1.28 ± 0.26 mmol/L, *p* < 0.01) and superoxide dismutase (SOD) (12.6 ± 3.71 U/mL vs. 15.4 ± 4.31 U/mL, *p* < 0.01). Similar, though less pronounced, improvements were also reported in the selenium group [34]. 

Alahmar et al. conducted a prospective controlled study with a 3-month follow-up including 50 patients with iOAT and 50 fertile men (controls) [35]. Patients received a daily dose of 200 mg of CoQ10. Semen analysis, seminal CoQ10 levels, ROS, TAC, CAT and sperm DNA fragmentation (SDF) as well as serum hormonal profile in patients after therapy were compared with the baseline values for patients and controls. Of note, CoQ10 therapy significantly increased total motility (*p* < 0.01), progressive motility (*p* < 0.05) and sperm concentration in iOAT men, compared to baseline. Results showed a significant decrease in fragmentation levels when infertile patients with iOAT were placed on CoQ10 therapy. It was also shown that treatment with CoQ10 substantially enhanced CAT, TAC and seminal CoQ10 levels in iOAT, and that total ROS levels decreased when compared with pretreatment values. These findings suggest that CoQ10 therapy could improve sperm parameters in infertile patients with idiopathic OAT.

More recently, a prospective study investigated 78 patients with idiopathic OA treated with 200 mg of CoQ10 for 6 months and 84 fertile men (controls) [36]. The authors found that treatment improved semen parameters and antioxidant measures and reduced SDF in infertile participants.

A summary of the studies evaluating CoQ10 monotherapy is presented in Table 1.

#### 3.1.2. CoQ10 Combination Therapy

In 2012, Busetto et al. were the first to prospectively evaluate a combination therapy including CoQ10. In total, 114 patients with iOAT were treated by a single daily dose of a formulation containing multiple antioxidant molecules (L-carnitine, acetyl-L-carnitine, fructose, citric acid, selenium, zinc, ascorbic acid, cyano-cobalamin, folic acid) and 20 mg CoQ10 for 4 months. Only sperm motility significantly increased after treatment (18.3 ± 3.8% to 42.1 ± 5.5%, *p* < 0.05) [37].

Abad et al. evaluated a commercial multivitamin comprising 20 mg CoQ10, L-carnitine, vitamin C, E, B9, B12, zinc and selenium. In a group of 20 asthenoteratozoospermic men, 3 months of therapy markedly improved type A motility, type A + B motility and vitality, whereas sperm density and normal morphology showed a minor rise (*p* = 0.042 and *p* = 0.04, respectively). Total and type B motility were unchanged [38].

In 2014, Kobori and colleagues conducted a larger, single-arm study employing a compound of CoQ10, vitamin C and E on 169 iOAT patients. Sperm concentration and motility significantly improved at 3 and 6 months. Sperm motility significantly improved at 3 and 6 months [39]. Moreover, during follow-up, 16 spontaneous pregnancies were achieved in this cohort.

A subsequent study with 20 patients with idiopathic asthenozoospermia evaluated the antioxidant capacity of a combination of CoQ10 (200 mg) and aspartic acid (2660 mg). After a 3-month treatment period, there was a significant improvement in sperm kinetics, but not in sperm count or in the number of atypical sperm cells. Levels of nitric oxide and peroxynitrite in seminal plasma decreased, whereas SOD activity increased. Moreover, the percentage of damaged DNA (assessed by comet assay) decreased significantly [40].

On the other hand, Gvozdjáková et al. observed an increase in sperm density in iOAT patients after 3 (39.8%, *p* < 0.001) and 6 months (78.0%, *p* < 0.001) of treatment with a multivitamin complex including carnitine, vitamin E and vitamin C and CoQ10 [41].

The effect of a combination of micronutrients (including 15 mg CoQ10 and carnitine) vs. carnitine alone was compared in a prospective, open-label, non-randomized study, by Lipovac et al. in infertile patients with at least one abnormal semen analysis. All the studied sperm parameters significantly improved after 3 months of treatment; however, in the combined micronutrient treatment group, the change of sperm density and progressive motility was higher [42].

In a 2018 double-blind, placebo-controlled trial, 77 infertile men with a high DNA fragmentation index (≥25%) were randomized to receive a commercial fertility supplement containing vitamins and antioxidants (including 10 mg CoQ10) or placebo twice a day for 6 months. After 3 months, the antioxidant group, compared to pre-treatment values, had higher sperm density (median: 24.4 × 10^6^/mL vs. 27.2 × 10^6^/mL, *p* = 0.028) [43].

In 2020, Terai et al. randomized 31 patients with idiopathic male infertility to receive an antioxidant supplement containing CoQ10 (90.26 mg), L-carnitine, zinc, astaxanthin, vitamin C, vitamin B12 and vitamin E or hochu-ekki-to (a Chinese herbal medicine). Sperm analysis and LH, FSH and testosterone serum concentrations were performed before and after 3 months of treatment. Both groups did not show any endocrinological or semen parameters increase. Total motile count was the only semen parameter to show a significant increase after treatment in the supplement group (*p* = 0.04), whereas no significant changes in hormonal parameters were observed [44].

Arafa et al. have recently evaluated the effect of antioxidant supplementation on conventional semen parameters and advanced sperm function tests in a population of infertile men [45]. A total of 148 subjects (119 in the idiopathic male infertility and 29 in the unexplained infertility group) received a 3-month treatment with three capsules twice a day of an antioxidant formula which provided, among others, 200 mg CoQ10 per day. Sperm analysis revealed an improvement in all parameters investigated, except for semen volume and sperm viability.

In 2020, Sadaghiani et al. enrolled, in their single-blinded trial, 50 oligospermic and asthenospermic patients, who were active smokers, to evaluate the effect of antioxidant supplementation (30 mg CoQ10, 8 mg zinc, 100 mg vitamin C, 12 mg vitamin E, 400 µg folic acid once a day and 200 mg selenium every other day) on semen parameters [46]. After 3 months of supplementation, mean sperm volume and total sperm count increased from 3.48 ± 1.44 to 3.71 ± 1.42 mL (*p* = 0.032) and from 21.76 ± 23.02 × 10^6^ to 23.22 ± 23.28 × 10^6^ (*p* = 0.001), respectively. Additionally, total sperm motility, progressive motility and normal sperm morphology increased from 27.22 ± 13.69% to 31.85 ± 5.82% (*p* = 0.001), from 9.82 ± 9.10% to 11.57 ± 10.18% (*p* = 0.001) and from 23.22 ± 23.28% to 33.60 ± 20.01% (*p* = 0.003), respectively [46].

The effect of a dietary supplement containing a mix of antioxidants and vitamins (including 40 mg CoQ10) versus placebo (1:1) was studied in the randomized, double-blind, placebo-controlled study by Kopets et al. [47]. Eighty-three patients with idiopathic male infertility were enrolled and randomized to receive a 6-month treatment. Normalization of semen analysis at 0, 2 and 4 months was the primary outcome, while the pregnancy rate was the secondary outcome. At 4 months, 69.0% of the patients in the treatment group and 22.0% in the placebo group had normal semen analysis (*p* < 0.001), whereas the pregnancy rate was significantly higher in the treatment than the placebo group (23.8% and 4.9%, *p* = 0.017) [47].

In 2021, Nazari et al. conducted an open study on 180 iOAT patients who received daily treatment of a combination of antioxidants (including 40 mg CoQ10) for 12 weeks. Sperm motility was unchanged, though sperm density (25 vs. 36 × 10^6^/mL, *p* = 0.004) and morphology (*p* = 0.01) improved [48].

Similarly, Gual-Frau et al. analyzed the impact of a multivitamin compound with 20 mg of CoQ10 in infertile men with grade I varicocele [49]. They found that only the total number of sperm increased after treatment.

More recently, Ma et al. [50] performed a single-blind RCT with patients randomly allocated to receive L-carnitine complex nutrient treatment (study group—15 g/bag, orally one bag at a time, twice a day, *n* = 73) or CoQ10 (control group—10 mg tablet orally, thrice daily, *n* = 70) with Vitamin E (100 mg tablet orally, thrice daily) for three months. They found that CoQ10 plus Vitamin E therapy resulted in improvement of sperm motility, morphology and testosterone levels. Conversely, L-carnitine significantly improves sperm motility, morphology and concentration, while also improving testosterone and LH levels.

A summary of the studies evaluating CoQ10 combination therapy is presented in Table 2.

#### 3.1.3. Effect of CoQ10 on Sperm DNA Fragmentation

Sperm DNA fragmentation (SDF) has progressively gained clinical importance in terms of reproductive outcomes both under natural and assisted reproductive technology (ART) conditions [51]. Several treatments of male infertility aim to reduce SDF values in order to improve the couple’s reproductive chance. While antioxidant therapy emerged as an effective option to improve semen parameters, its impact on SDF is still a matter of debate [52]. Here, we analyzed the literature with the specific focus of the effect of CoQ10 on SDF.

Antioxidant treatment was found to be effective in reducing SDF values in a study by Nadjarzadeh and colleagues [32]. Furthermore, in the subgroup with increased pretreatment SDF levels, supplementation increased the success rate of intracytoplasmic sperm injection (ICSI) [32].

Similarly, two years later, Abad et. al. showed that a multicomponent antioxidant treatment could significantly diminish the progression of sperm DNA fragmentation over increasing incubation time; moreover, the proportion of sperm cells bearing degraded DNA was significantly reduced (7.32 ± 4.12% vs. 5.66 ± 3.21%, *p* = 0.04) [38].

In 2015, Gual-Frau et al. investigated the effect of a multivitamin compound containing CoQ10 in infertile patients affected by varicocele and with high SDF levels. Three months of treatment lead to a 22.1% decrease in SDF (*p* = 0.02) and a 31.3% reduction in DNA-degraded sperm cells (*p* = 0.07) [49]. 

In a subsequent study, Alahmar et al. administered a 3-month course of 200 mg CoQ10 to 65 patients affected by idiopathic OA related infertility and 40 fertile subjects. The results showed an improvement in semen parameters and a reduction in OS markers and SDF in the former group [53].

On the other hand, an RCT from 2018 by Stenqvis et al. failed to confirm these findings. Seventy-seven infertile men received a compound containing, among others, 10 mg CoQ10 for 6 months. At the end of the treatment phase SDF and other sperm parameters were comparable between groups, although sperm density did rise in the antioxidant group compared to pre-treatment [43].

More recently, Arafa et al. conducted a trial categorizing subjects with idiopathic (119 patients) and unexplained male infertility (29 patients). The 148 subjects were administered a course of a mixed supplement composed of 200 mg CoQ10 and other antioxidants. In the first group, 3 months of treatment lead to an improvement in SDF and all semen parameters (with the exception of sperm volume and viability). On the other hand, SDF was stable in the unexplained infertility group [45].

As previously reported, Alahmar et al. investigated 50 patients with iOAT treated with a daily dose of 200 mg of CoQ10 and 50 fertile men (controls) [35]. They found a significant decrease in SDF levels after treatment in infertile men (38.6% ± 7.9 vs. 34.5% ± 9.3, *p* < 0.001).

#### 3.1.4. CoQ10: Effect on Natural Pregnancy and ART

Different authors have described an increase in pregnancy rates after CoQ10 administration [54,55] and this has been attributed to its positive effect on sperm concentration and motility. For instance, both Abad and Kobori have reported natural pregnancies after a combined antioxidant treatment which improved semen parameters [38,39].

Similarly, Kopets and colleagues studied the effect of a dietary supplement containing a mix of antioxidants and vitamins (including 40 mg CoQ10) in a 1:1 RCT. Four-month normalization of semen parameters was significantly more frequent in the treatment group (69.0 vs. 22.0%, *p* < 0.001), as it was for pregnancy rate (23.8% and 4.9%, *p* = 0.017) [47].

In a prospective series published in 2012, Safarinejad et.al. enrolled 287 iOAT patients to be administered a 12-month course of daily 300 mg CoQ10. Patients were then kept on follow up for an additional year. Mean treatment to pregnancy time was 8.4 ± 4.7, with a total pregnancy rate of 34.1%; semen parameters were linearly associated with the latter [56].

Again, focusing on combination therapy, Gvozdjáková and colleagues investigated the efficacy of a mixed compound of carnitine, CoQ10 and vitamin C and E on OA-related infertility. After treatment, a 45% overall pregnancy rate, 7.5% of which through ART, was reported [41].

As previously reported, Alahmar et al. [36] investigated the effect of 200 mg CoQ10 for 6 months in 78 iOA infertile men vs. controls. Treatment significantly improved semen parameters, antioxidant biomarkers and SDF. The pregnancy rate was 24.2%, and time to pregnancy (TTP) was 20.52 ± 6.72 months following 6 months of CoQ10 therapy and another 18 months of follow-up. CoQ10 level, male age, semen parameters and ROS levels were found to be predictors of pregnancy outcomes and TTP.

### 3.2. Melatonin

#### 3.2.1. Melatonin: Role in the Testis

In addition to its well-known effect on daily and annual rhythmicity, melatonin has been found to regulate reproductive function via modulating the release of gonadotropin-releasing hormones (GnRH) [57]. Furthermore, melatonin is absorbed by the testis, where it affects testicular function directly [58,59] by acting on receptors present on Leydig [60], Sertoli [61,62] and intratesticular inflammatory [63] cells. Intriguingly, melatonin and testosterone share comparable circadian rhythms, and in animal species, this hormone influences various aspects of testicular function to facilitate seasonal reproduction [64,65]. Specifically, melatonin was found to inhibit GnRH-induced testosterone release [60,66,67] and to stimulate the testicular conversion of testosterone into dihydrotestosterone (DHT) [68]. Subsequent studies demonstrated that this may occur via a local corticotropin-releasing hormone (CRH) system, all components of which were then identified in the testes of infertile men [67]. This indolamine also protects the testis from local inflammatory processes and ROS. Consequently, melatonin exerts anti-proliferative and anti-inflammatory effects on testicular macrophages [69] and mast cells [63], and its testicular concentrations demonstrated a negative correlation with the concentration of pro-apoptotic molecules [63] and the number of macrophages [70] in biopsies from infertile patients. In addition, melatonin functions as a free radical scavenger [71], preventing apoptosis and restoring testicular function [72,73,74]. Accordingly, melatonin treatment reduces the severity of induced testicular damage in animal models [75,76,77,78,79,80,81]. Lastly, the hormone acts on Sertoli cells by enhancing their responsiveness to follicle stimulating hormone during development [82] and by inducing the secretion of growth factors active on spermatogonial stem cells proliferation [83,84,85].

Few clinical studies have investigated the impact of melatonin treatment on semen parameters (Table 3). Awad and colleagues investigated a possible correlation in both fertile and infertile men, categorizing the latter group by semen analysis alterations. The authors observed lower melatonin levels in men affected by non-obstructive azoospermia, in those displaying impaired sperm motility and leucocytospermia (the levels of whom were lowest) [86]. In 2016, Kratz et al. measured seminal plasma concentrations of melatonin and oxidative stress parameters in fertile normozoospermic and infertile terato- or azoospermic infertile subjects. The authors report higher levels of seminal plasma melatonin in normozoospermic fertile men, with no difference between different subgroups of infertile men. Moreover, infertile subjects displayed increased concentrations of oxidation products in seminal fluid, with azoospermic patients displaying the highest levels [87]. The study once again suggests the possible relation between melatonin and oxidative stress in the infertile male.

The potential clinical benefit of melatonin supplementation on semen parameters was tested after varicocele treatment by Lu and colleagues [88]. Fifty-four infertile, mildly oligospermic men affected by varicocele were randomized to receive either 400 mg of melatonin or placebo after varicocele treatment. At 3 and 6 months, the patients treated by melatonin showed a stronger improvement in terms of semen parameters (sperm concentration, motility and proportions of normally formed spermatozoa), peripheral blood inhibin B levels and total antioxidant compared with the placebo group.

#### 3.2.2. Melatonin in ART Outcomes

The role of melatonin on female reproduction has been more extensively studied. For instance, in a systematic review and meta-analysis from 2020, Hu and colleagues described the role of melatonin in ART. The authors concluded that melatonin is indeed associated with higher embryo quality and clinical pregnancy rate, though not with a more significant live birth rate [27]. Besides these findings, which are beyond the scope of this review, in this context, melatonin was shown to act, among other mechanisms, on inflammation, apoptosis and oxidative stress modulation [89,90]. These observations, thus, strengthen the ones made on male reproductive system, thus confirming this hormone’s potential.

## 4. Discussion

Supplementation therapy has been widely employed in the context of infertility as a mean to improve the success rate in achieving pregnancy both spontaneously and by means of ART. In this context, the evidence strongly suggests that oxidative stress plays a central role in idiopathic infertility [15,16,17], thus raising interest towards antioxidant therapy in this field.

Coenzyme Q10 is involved in mitochondrial energy production and has a key role as an antioxidant for cell membranes and lipoproteins. For this reason, different authors have raised interest towards its use in the field of reproductive medicine. As discussed in this review, CoQ10 monotherapy was shown to enhance sperm motility and concentration as well as improving sperm DNA fragmentation in infertile men. Moreover, the results also suggest that CoQ10 may itself be effective in improving conception rates. The improvement in semen parameters reported by many authors was then followed by a higher frequency of spontaneous pregnancy and better outcomes for ART. Nonetheless, it must be noted that these were mostly assessed as secondary outcomes, and one should be careful in interpreting these results. In fact, despite single studies suggesting a positive effect of CoQ10 on pregnancy outcomes, a previous systematic review and meta-analysis revealed that there is no evidence that CoQ10 increases either live birth or pregnancy rates, but there is a global improvement in sperm parameters [91].

Similar to other molecules, CoQ10 use in male infertility showed premising results, but studies are of low evidence, mainly with small sample size and with a wide range of dosage and timing of administration, thus precluding its widespread use [92]. In particular, it must be noted that results from studies dealing with combination therapy are quite heterogeneous. First, the dose of CoQ10 in these studies is generally lower (range 10–200 mg) compared to that investigated in monotherapy (200–300 mg); second, the presence of multiple antioxidant agents makes hard the interpretation of which is the real responsible for the biological effect; finally, the duration of treatment was not standardized. As a whole, caution should be used when interpreting results from studies dealing with antioxidant cocktails.

Similarly, we analyzed the existing evidence concerning the role of melatonin in male infertility, although the data regarding this molecule is limited. Besides its role as a free radical scavenger, and thus, its antioxidant potential, which might itself justify its employment, melatonin might have a more complex role. Animal models have in fact shown that this molecule may contribute to gonadal physiology by modulating androgen production (both centrally and locally), by acting as an immunomodulatory compound and by influencing the progression of germ cells to spermatozoa. Clinical data in men are scarce, whereas there is wider evidence regarding its efficacy on female fertility from ovarian aging to clinical improvement on in vitro fertilization success rate. In summary, preclinical data regarding melatonin show potential in the context of male infertility, though clinical studies are needed in order to assess the concrete possibility of its employment.

As previously mentioned, in an attempt to counteract the deleterious effects of OS, urologists resorted to antioxidant supplementation. Considering that indiscriminate and empirical use of these compounds can exert detrimental effects through a reduction state, physicians need to be aware of the “antioxidant paradox” [93]. The latter was termed by Halliwell et al. to describe that the overuse of antioxidants has no preventative or therapeutic effect at all. In this context, Symeonidis et al. [94], in a recent review, highlighted the need for redox balance, thus safeguarding redox homeostasis. This article signifies that moderation is the key to optimal sperm regulation.

## 5. Conclusions

In recent years, a growing body of literature has shown that an altered redox balance in seminal fluid may display deleterious effects on sperm homeostasis, leading to male infertility [95,96]. The present narrative review shows the antioxidant properties of CoQ10 and its beneficial role on semen parameters and sperm DNA fragmentation. Moreover, CoQ10 administration in couples resulted in improved ART outcomes, such as increased fertilization rates in IVF/ICSI.

Further in-depth interventions are needed to reveal the exact mechanism of action of CoQ10 and to determine the appropriate standardized dose and duration of CoQ10 supplementation in the treatment of specific male infertility cases. Additional evidence is needed to further support the treatment of male infertility with melatonin supplementation.

## Figures and Tables

**Table 1:** Summary of studies evaluating CoQ10 monotherapy. TAC: Total antioxidant capacity; CAT: catalase; SOD: superoxide dismutase. FSH = Follicle stimulating hormone; LH = Luteinizing hormone.

Group	Number of Patients	Therapy Administrated	Main Results
Balercia et al., [28]	28	CoQ10 200 mg/day for 6 months	Increase in total and forward motility Increased levels of CoQ10 in sperm cells and seminal plasma
Safarinejad et al., [29]	106	CoQ10 300 mg/day for 6 and half months	Higher total sperm counts and motility Increase in frequency of acrosome reaction Increase in inhibin B levels and a decrease in FSH and LH levels
Safarinejad et al., [30]	228	114 CoQ10 (ubiquinol) 200 mg/day for 6 and half months 114 placebo	Increase in sperm density, motility and morphology Increase in inhibin B levels and a decrease in FSH and LH serum levels
Festa et al., [31]	38	CoQ10 100 mg/day for 3 months	Increase in sperm density and forward motility Increase in total antioxidant capacity
Nadjarzadeh et al., [32]	47	CoQ10 200 mg/day for 3 months	Increase in total antioxidant capacity of seminal plasma. No difference in semen parameters between the CoQ10 and placebo groups
Alahmar et al., [33]	65	CoQ10 200 mg/day for 6 months CoQ10 400 mg/day for 6 months	In both groups, increase in sperm concentration and progressive and total motility In both groups, increase in TAC, CAT and SOD activity Stronger improvement with higher dosage
Alahmar et al., [34]	35	CoQ10 200 mg/day for 3 months	Increase in sperm concentration, progressive sperm motility and total sperm motility Improvement in TAC, SOD and CAT
Alahmar et al., [35]	50	CoQ10 (ubiquinol) 200 mg/day for 3 months	Increase in sperm concentration, progressive sperm motility and total sperm motility
Alahmar et al., [36]	78	CoQ10 (ubiquinol) 200 mg/day for 6 months	Increase in semen volume, sperm concentration, progressive sand total perm motility and normal morphology

**Table 2:** Studies evaluating CoQ10 in combination with other supplements. ROS: reactive oxygen species; SOD: superoxide dismutase; SDF: Sperm DNA fragmentation; ORP: oxidation reduction potential.

Group	Patients	Therapy Administrated	Main Results
Busetto et al., [37]	114	L-carnitine, acetyl-L-carnitine, fructose, citric acid, selenium, zinc, ascorbic acid, cyano-cobalamin, folic acid and 20 mg CoQ10 daily for 4 months	Increase in sperm motility
Abad et al., [38]	20	1500 mg L-carnitine, 60 mg vitamin C, 10 mg vitamin E, 200 µg vitamin B9, 1 µg vitamin B12, 10 mg zinc, 50 mcg selenium and 20 mg CoQ10 daily for 3 months	Improvement in type A + B motility, vitality, sperm density and normal morphology Reduction in DNA degraded sperm
Kobori et al., [39]	169	80 mg vitamin C and 40 mg vitamin E and 120 mg CoQ10 daily for 6 months	Increase in sperm motility and sperm concentration
Tirabassi et al., [40]	20	Aspartic acid 2660 mg and 200 mg CoQ10 daily for 3 months	Improvement in sperm kinetics and increase in SOD activity Decrease in ROS levels in seminal plasma Correlation of seminal fluid oxidation state and DNA damage index
Gvozdjáková et al., [41]	40	440 mg L-carnitine fumarate, 75 IU vitamin E, 12 mg vitamin C and 30 mg CoQ10 daily for 6 months	Increase in sperm density Increase in seminal fluid CoQ10 concentration Decreased oxidative stress parameters
Lipovac et al., [42]	143 group 1	440 mg L-carnitine, 250 mg L-arginine, 40 mg zinc, 120 mg vitamin E, 80 mg glutathione, 60 μg selenium, 800 μg folic acid and 15 mg CoQ10 daily for 3 months VS 500 mg l-carnitine/twice a day alone	Improved semen parameters in the multivitamin group
	156 group 2		
Stenqvist et al., [43]	37 group 1	Vitamin C 30 mg, vitamin E5 mg, vitamin B12 0.5 lg, l-carnitine 750 mg, and folic acid 100 lg, zinc 5 mg, selenium 25 lg, with maltodextrin, calcium carbonate, citric acid, steviol glycoside, flavors, beta-carotene, silicon dioxide and coenzyme Q10 10 mg twice/daily VS placebo for 6 months	Increased sperm density in group 1 No differences in semen parameters or SDF
	40 group 2		
Terai et al., [44]	15 group 1	L-Carnitine 750.1 mg, Zinc 30 mg, Astaxanthin 16.05 mg, Vitamin C 1000 mg, Vitamin B12 60.1 μg, Vitamin E 150 mg and CoQ10 90.26 mg vs. Chinese herbal medicine hochu-ekki-to (HE), 3 times/daily for 3 months	No difference in endocrinological or semen parameters In group 1 increase of total motile count
	16 group 2		
Arafa et al., [45]	148 group 1 idiopathic infertility	Vitamin C, 120 mg vitamin D3, 1200 IU vitamin E (as mixed tocopherols), 200 IU vitamin K, 80 µg thiamin, 3 mg, riboflavin, 3.4 mg niacin, 20 mg vitamin B6, 25 mg folate, 800 µg vitamin B12, 1000 µg biotin, 600 µg pantothenic acid, 20 mg iodine, 150 µg zinc, 30 mg selenium, 140 µg copper, 1 mg manganese, 2 mg chromium, 120 µg molybdenum, 75 µg l-carnitine tartrate, 2000 mg L-arginine, 350 mg CoQ10, 200 mg N-acetyl L-cysteine, 200 mg grapeseed extract, 20 mg lycopene, 10 mg and benfotiamine 1 mg daily for 3 months	Group 1: Decrease in SDF values and ORP. Improvement of sperm parameters Group 2 Improvement in progressive motility only
	29 group 2 unexplained infertility		
Sadaghiani et al., [46]	50	30 mg CoQ10, 8 mg zinc, 100 mg vitamin C, 12 mg vitamin E, 400 µg folic acid once a day and 200 mg selenium every other day for 3 months	Increase in mean volume of sperm and total sperm count Increased total sperm motility, progressive motility and normal sperm morphology
Kopets et al., [47]	42 group 1 supplementation	L-carnitine/L-acetyl-carnitine 1990 mg, L-arginine 250 mg, glutathione 100 mg, co-enzyme Q10 40 mg, zinc 7,5 mg (75% of recommended daily allowance, RDA), vitamin B9 234 µg (117% RDA), vitamin B12 2 µg (80% RDA), selenium 50 mcg (91% RDA), excipients sorbitol, maltodextrin, orange/beta-carotene colorant, saccharide, acesulfame potassium, and silicium dioxid vs. placebo daily for 6 months	69.0% vs. 22.0% normal semen analysis in study group vs. placebo 23.8% vs. 4.9% spontaneous pregnancies in study group vs. placebo
	41 group 2 placebo		
Nazari et al., [48]	180	1500 mg of L-Carnitine, 60 mg of vitamin C, 20 mg of coenzyme Q10, 10 mg of vitamin E, 10 mg of zinc, 200 µg of vitamin B9, 50 µg of selenium, 1 µg of vitamin B12 twice/daily for 3 months	Increase in sperm density and morphology
Gual-Frau et al., [49]	20	1500 mg L-Carnitine, 60 mg vitamin C, 20 mg coenzyme Q10, 10 mg vitamin E, 200 μg vitamin B9, 1 μg vitamin B12, 10 mg zinc, 50 μg selenium	Increase in total number of sperm, but other semen parameters were unaffected
Ma et al., [50]	70	CoQ10 10 mg tablet orally, thrice daily plus Vitamin E 100 mg tablet orally, thrice daily, for three months.	Improvement in sperm motility, morphology and testosterone levels

**Table 3:** Studies evaluating the association between melatonin and semen parameters. OA = Oligoasthenozoospermia; NOA = Non-obstructive azoospermia.

Group	Number of Patients	Main Results
Awad et al., 2006	Fertile normozoospermic men (*n* = 20) OA (*n* = 20) OA with leucocytospermia (*n* = 20) OA with varicocele (*n* = 20) NOA with high FSH (*n* = 20) NOA with normal FSH (*n* = 20)	Serum and seminal plasma melatonin levels in all infertile groups were reduced significantly compared with their levels in the fertile group OA with leucocytospermia had the lowest levels of melatonin Melatonin was positively correlated with sperm motility
Kratz et al., 2016	azoospermia (*n* = 37) theratozoospermia (*n* = 29) fertile controls (normozoospermia, *n* = 37)	Melatonin was lower in azoospermic (*p* < 0.0001) and theratozoospermic (*p* < 0.0001) patients versus fertile men
Lu et al., 2018	27 infertile men receiving 400 mg of melatonin for 3 months and 27 receiving placebo for 3 months after varicocelectomy	Sperm concentration, motility and proportions of normally formed spermatozoa significantly improved in melatonin group compared with placebo group

## Data Availability

Not applicable.

## Supplementary Materials

The following supporting information can be downloaded at: https://www.mdpi.com/article/10.3390/nu14214585/s1, Table S1: Narrative Review Checklist.
