# Supplementary figures and images for: Coenzyme Q10 and Melatonin for the Treatment of Male Infertility: A Narrative Review
Source: Nutrients. 2022 Nov 1;14(21):4585. doi: 10.3390/nu14214585 (PMC9658523; doi:10.3390/nu14214585)

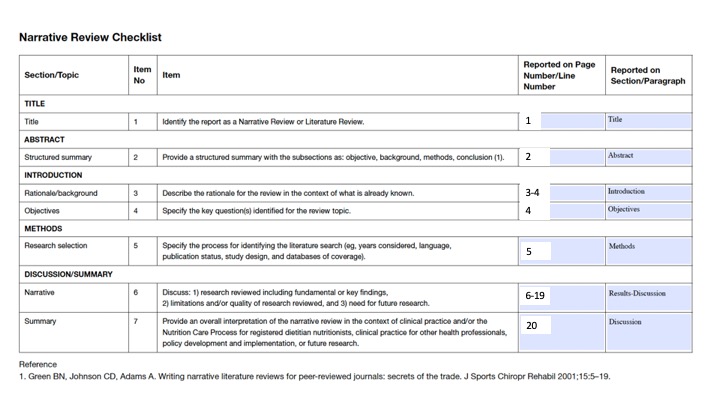

Supplement: Supplementary file 1 [file nutrients-14-04585-s001.zip › nutrients-1959786-supplementary.jpg]
